# Editorial: Methods and applications in cellular neuropathology

**DOI:** 10.3389/fncel.2023.1244414

**Published:** 2023-07-11

**Authors:** Gabriela O. Bodea, Muge Yemisci

**Affiliations:** ^1^Queensland Brain Institute, University of Queensland, Brisbane, QLD, Australia; ^2^Institute of Neurological Sciences and Psychiatry, Department of Basic Neurosciences and Psychiatry, Faculty of Medicine, Department of Neurology, Hacettepe University, Sihhiye, Ankara, Türkiye; ^3^Neuroscience and Neurotechnology Center of Excellence (NÖROM), Ankara, Türkiye

**Keywords:** neuroscience methodology, neuropathology, cellular neuroscience, viral vector, regenerative medicine, molecular profiling of brain cells, neurovascular unit (NVU) model, emerging approaches

The development of tools and applications in cellular neuroscience research has rapidly advanced over the past decade and opened new avenues of research. In this Research Topic, we showcase the latest methods and analyses that yield exciting advancements in cellular neuropathology. We highlight techniques used to label and manipulate specific brain cell types, molecular profiling of low abundance cells, improvements in regenerative medicine protocols and *in vitro*-based assays to test therapeutics. Together, these applications will broaden our understanding of brain functions at the cellular level and in health and disease.

## Enriching the toolbox of viral vector techniques to target specific brain cells and areas

The adeno-associated viral (AAV) vectors offer a powerful tool for labeling, monitoring, and modulation of brain cell activity in a cell-type-specific fashion. Dorgans et al. generated and validated novel AAV vectors to target the inferior olivary nucleus (ION) neurons. The authors applied custom AAV vectors to the ION of the cerebellum, an area that gates essential timely-related signals to the cerebellum for motor control. This new tool is helpful for the explicit labeling of ION cells, and it allows calcium imaging approaches to monitor even subtle activity signatures in specific cell types *in vitro* and *in vivo*. This warrants a more detailed investigation into the circuits and connectivity of the olivo-cerebellar system *in vivo* under normal conditions and pathology.

## Novel approaches for molecular profiling of brain cells

The latest technical advances used in Xu et al. and Ji et al. bring high sensitivity detection and transcriptome profiling of low abundance cells and protein posttranslational modifications.

Single-cell RNA sequencing has been extensively used in the past years to profile brain cells, providing unprecedented knowledge of cell heterogeneity and vulnerability to different neurological pathologies, regulatory interactions, and biomarkers (Piwecka et al., 2023). The advantage of such an approach shines when cells are in low amounts, vulnerable to isolation protocols, and typically lost in bulk analyses. Xu et al. have applied 10x Genomics single-cell RNA sequencing to cochlear sensory epithelial cells embedded in the temporal bone, both in low number and challenging to profile. Here, the authors characterized the transcriptome of various cell types, including hair cells, supporting cells, and immune cells in the juvenile and mature cochlea, and have mapped genes related to pathology, such as deafness genes, to corresponding cell types. This technique also allowed identifying novel long non-coding RNAs expressed at different developmental stages opening new directions of research. The study provides an invaluable resource for cochlear maturation at single-cell resolution.

Hard-to-trace protein modifications (such as lysine acetylation) are involved in brain development and the pathophysiological mechanisms of neurodegenerative diseases (Lilja et al., 2013; Park et al., 2016). In the study within our Research Topic, Ji et al. have profiled and characterized the brain acetylome in mice. The authors have identified brain-specific acetylated proteins and constructed an acetylome map which may provide a valuable basis for future research into acetylome dynamics in various pathological conditions.

## Improving methodology in nervous system regenerative medicine

Over the past few decades, much research has focused on promoting axonal regeneration, replenishing brain cells, and rebuilding lost pathways after nervous system injuries and neurodegenerative conditions. A growing body of evidence suggests that multipotent stromal cells or mesenchymal stem cells (MSC), derived from adipose tissue (A-MSC) or bone marrow (BM-MSC), are strong candidates for nervous system regeneration due to their potential for neuronal differentiation and the safety of transplantation (Petrenko et al., 2020). However, as explored in this Research Topic by González-Cubero et al. and Hashemizadeh et al., MSCs-based therapies require further optimisation in culturing, delivery routes, timing, and validation of their efficacy in various pathological contexts. Both A-MSC- and A-MSC-derived conditioned mediums efficiently promote myelination and regeneration of axons in a rat model of tumor necrotic factor (TNF) sciatic nerve insult, with potential applications in neuropathic pain. In spinal cord injury, a combination therapy involving modulation of local inflammation and BM-MSC transplantation may improve motor survival and help myelination.

## Cell culture-based models to mimic biology that is favorable to studying pathophysiological mechanisms of diseases and the delivery of therapeutics

Cell culture models mimicking the cellular interactions of the brain cells are used to improve our understanding of structures, functions, and environment at the microscopic level. Significant progress was made in the two-dimensional and three-dimensional models over the past decades. However, more work is needed to answer essential mechanistic questions and efficiently test therapeutics in preclinical studies in complex neurological diseases, including neurodegeneration (Cetin et al., 2022). One well-established feature in neurodegenerative conditions is microglial cell activation resulting in the release of pro-inflammatory cytokines. In this Research Topic, De Chirico et al. provide a characterization of the human microglia clone 3 (HMC3) cell line and propose its use for modeling Parkinson's disease-like neuroinflammatory profile *in vitro*.

The neurovascular unit (NVU) concept emerged in 2001 and refers to the relationship between brain cells and blood vessels in the maintenance of the blood-brain barrier (BBB) and regulation of blood supply to the brain. NVU dysfunctions have been recently associated with most neurodegenerative diseases (Iadecola, 2017; Yu et al., 2020). Here, Barberio et al. described a novel culturing system consisting of co-culturing endothelial cells, astrocyte cells, and neuronal cells, which allows for the study of barrier functions of the NVU. As most drugs cannot pass the BBB, the authors highlight the future avenues for drug testing and delivery using this co-culturing system.

Environmental factors, such as organophosphates used in pesticides, can inhibit acetylcholinesterase (AChE) that catalyzes the breakdown of acetylcholine neurotransmitters and is essential for the termination of signal transmission. AChE inhibition, therefore, results in increased cholinergic signaling leading to several dysfunctions in both the peripheral and central nervous system, such as disruption of voluntary movement, loss of consciousness and seizure. Thinschmidt et al. have developed an improved brain slice assay that can be used as a preclinical model for quantification of the ability of known and novel AChE reactivators to restore the effects of native AchE in the amygdala, hippocampus or other brain areas.

This Research Topic highlights a small sample from the broad range of emerging approaches that enable discoveries in cellular neuropathology. We want to share our excitement with the readers and witness novel directions that these methods will bring in the near future.

## Author contributions

GB drafted, edited, and reviewed the manuscript. MY edited and reviewed the final manuscript. All authors contributed to the article and approved the submitted version.
